# Forecasting major impacts of COVID-19 pandemic on country-driven sectors: challenges, lessons, and future roadmap

**DOI:** 10.1007/s00779-021-01530-7

**Published:** 2021-03-26

**Authors:** Saket Kumar, Rajkumar Viral, Vikas Deep, Purushottam Sharma, Manoj Kumar, Mufti Mahmud, Thompson Stephan

**Affiliations:** 1 Amity School of Engineering and Technology, Amity University Uttar Pradesh, Noida, India; 2 School of Computer Science, University of Petroleum, Energy Studies (UPES), Bidoli, Dehradun, India; 3 Department of Computer Science, Nottingham Trent University, Clifton Campus, Clifton, Nottingham, NG11 8NS UK; 4 Medical Technologies Innovation Facility, Nottingham Trent University, Clifton Campus, Clifton, Nottingham, NG11 8NS UK; 5 Department of Computer Science and Engineering, Faculty of Engineering and Technology, M. S. Ramaiah University of Applied Sciences, Bangalore, Karnataka India

**Keywords:** Corona virus, Mathematical modelling, COVID-19 impact, Forecast affect

## Abstract

The pandemic caused by the coronavirus disease 2019 (COVID-19) has produced a global health calamity that has a profound impact on the way of perceiving the world and everyday lives. This has appeared as the greatest threat of the time for the entire world in terms of its impact on human mortality rate and many other societal fronts or driving forces whose estimations are yet to be known. Therefore, this study focuses on the most crucial sectors that are severely impacted due to the COVID-19 pandemic, in particular reference to India. Considered based on their direct link to a country’s overall economy, these sectors include economic and financial, educational, healthcare, industrial, power and energy, oil market, employment, and environment. Based on available data about the pandemic and the above-mentioned sectors, as well as forecasted data about COVID-19 spreading, four inclusive mathematical models, namely—exponential smoothing, linear regression, Holt, and Winters, are used to analyse the gravity of the impacts due to this COVID-19 outbreak which is also graphically visualized. All the models are tested using data such as COVID-19 infection rate, number of daily cases and deaths, GDP of India, and unemployment. Comparing the obtained results, the best prediction model is presented. This study aims to evaluate the impact of this pandemic on country-driven sectors and recommends some strategies to lessen these impacts on a country’s economy.

## Introduction

Coronavirus disease-19 (COVID-19), a journey of slayer, started in a city of 11 million people named Wuhan, located in China. On 31 December 2019, the first case of unusual pneumonia was detected in Wuhan city. Within five days of the period, China alerted the World Health Organization (WHO) about this unusual pneumonia because, within five days of the period, the infected cases increase up to 40. On January 7, 2020, the WHO officially announced that the virus spread in the Wuhan city is called as SARS-nCov-2, which is related from corona family. And it includes SARS and commonly has cold symptoms [1]. Finally, on January 11, China announces his first death from this virus. The virus was spreading rapidly in China, and on 13 January 2020, the first country Thailand outside the China reported the first case of this coronavirus. The reason of the patient given by Thailand that she/he travels from Wuhan city. This was the first evidence that this virus can spread from human to human. On January 17 and 2020, China announces second and third death from COVID-19. At that time, China declare that more than 200 infected peoples are there. Finally, China announces lockdown on 23 January 2020 [2]. The symptoms of coronavirus and human-to-human transmission of this virus have been proved to date. End of the month of January 2020, the WHO declared a global emergency due to this virus and many countries like India, Philippines, Russia, Spain, Sweden, and the UK, Australia, Canada, Germany, Japan, Singapore, the USA, the UAE, and Vietnam reported first case in this week of January 2020. At the end of the month of January 2020, the cases increase rapidly in China and until 7 Feb 2020, 908 deaths claimed by China with 40,171 infected. Drastically, the ratio increases in China even after lockdown. China also declared that, the symptoms of corona virus has been shown in human within 5 to 10 days. This is the reason, that after lockdown, the number of cases increases in China because until 23 January 2020, it already spread in the community rapidly. The numbers of the infected people increased exponentially throughout the world. During 13–14 Feb 2020, Japan, France, Taiwan, and Egypt encountered its first case [3]. Until 17 Feb, 73,332 infected people encountered globally with death of 1873 people. The majority of infected people were from China to date. This is the most mysterious week in the history of coronavirus. This week the coronavirus spread globally and reach most of the countries of the world, including Kuwait, Bahrain, Iraq, Oman, Qatar, Norway, Romania, Greece, Georgia, Pakistan, Afghanistan, North Macedonia, Brazil, Estonia, Denmark, Northern Ireland, Netherlands, Lithuania, and Wales. Including gulf countries and Asian countries, this virus attacked already. This was the time, when most of the countries start thinking about lockdown. On March 02, 2020, 90,443 infected were encountered with death of 3117. Between 09 March 2020 and 15 March 2020, Lebanon, Morocco, Kazakhstan, Philippines, and Austria announce complete lockdown in the country and people advised to stay at home for being safe. The number of infected people increases rapidly and until the mid of March 2020s, the number of infected globally increases up to 182,429 with 7170 deaths. The number of deaths also increased with time. WHO claims that coronavirus attacks on the persons’ immune system, and it declares that most of the death cases are infected from any disease like blood pressure, heart disease, etc. On March 25, 2020, the USA declared 124,000 infected with 2000 deaths but in few days, the numbers increase to 300,000 infected with death of 4000 people. India and South Africa declared complete lockdown on 27 March 2020. The decision of lockdown in India was difficult for the current government due to higher population but government has taken this decision [4].

**Fig. 1:**
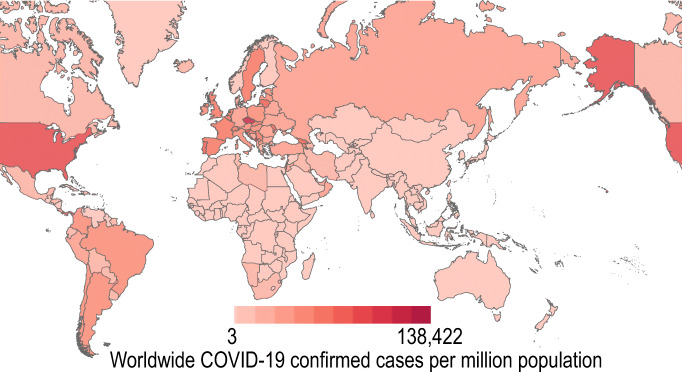
COVID-19 spread worldwide

**Fig. 2:**
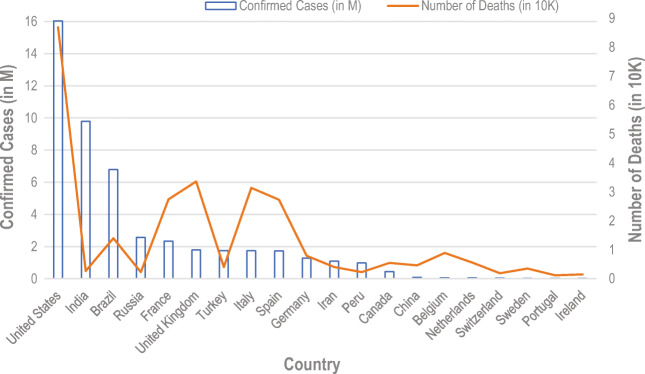
Top 20 countries that are affected by COVID-19

In four and half months, the coronavirus spread globally and infected 4,527,290 people, and took the life of 303,418. In the last several decades, this virus was most dangerous and a complete slayer for human. In May 2020, the virus attacked several countries, and most of the countries in the world came under this virus. Figure 1. shows the global spread of COVID-19 virus until 14 May 2020 [4]. The dark brown colour in Fig. 1. represents more than 1 million people infected. The most affected countries at present until 14 May 2020 are the USA, Spain, Italy, France, Germany, UK, Turkey, Iran, China, Russia, Brazil, Belgium, Canada, Netherlands, Switzerland, Portugal, India, Peru, Ireland, Sweden, etc. These are top 20 countries in the world which are affected badly from this killer virus. Figure 2. illustrates the seriousness of COVID-19 in these top 20 countries [4].

Despite several computer-aided systems to tackle the problem using artificial intelligence [5] and machine learning [6–11], the control of the virus has been very slow. The death in the United States of America is at the peak. Most of the infected cases are in the USA. Spain, Italy, and France are the countries that suffer after the USA. These countries death rate is also too high. China is on saturation until the date 11 Dec 2020. The cases in China reduce and the economy started again from lockdown.

**Fig. 3:**
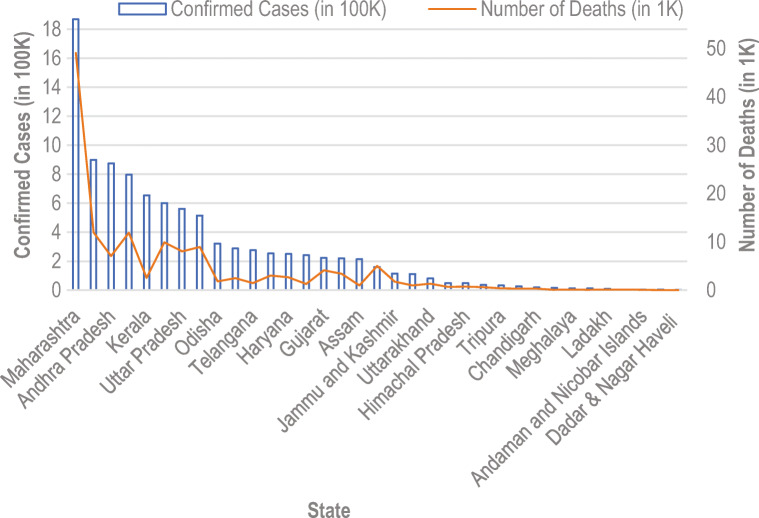
State wise analysis of India for the confirmed cases and number of deaths

In this list, India is at second position in top 19 countries in affected number of cases. With huge population of 135.26 crores, India controlled COVID-19 very well. Until Dec 11, 2020, according to the Ministry of Home and family Welfare [12], Gov. of India declare 9,796,992 infected cases with 142,222 deaths. The cured patient numbers also increase in India. Until 11 Dec 2020, 9,290,834 infected people cured and go to home safely. Few states like Maharashtra, Gujarat, Delhi, Rajasthan, Tamil Nadu, Madhya Pradesh, Uttar Pradesh, and Telangana are the states which encountered more than thousand cases. Maharashtra encountered 1,868,172 infected cases alone. Gujarat and Delhi encountered more than 223,919 infected. Tamil Nadu, Madhya Pradesh, Uttar Pradesh, and Telangana are the states more than 795,240 infected. Figure 3 depicts the whole scenario of India until 11 Dec 2020 [12].

As per Fig. 3, the number of deaths in Maharashtra is more significant than in other states, but the number of cured patients is also more in these states. In January and February 2020, only 3 cases were encountered in India, and then in March, the number of cases rapidly increase. At the end of March 2020, India’s central government announces a complete lockdown till 17th May 2020. The entire country gets a total lockdown, and the government applies several rules to follow this lockdown.

On the other hand, most of the countries already declared complete lockdown, due to which most of the countries suffer from huge economical loss. United Nations releases emergency funds of US$15 million to help different countries to fight with this coronavirus [13]. The WHO release a report of strategic preparedness and response plan for every country and also release preparedness and response status of different countries, territories, and areas. WHO distribute all the countries in five different levels and two categories. The categories are community transmission and greater than or equal to 10 cases per day. According to WHO, Canada, Switzerland, the USA are in level 5 and in category of community transfer. According to the WHO report, if the virus COVID 19 reaches to community transfer state, then the number of cases will exponentially increase like a chain reaction and cannot be controlled very soon [14]. The countries which already reach at the state of community transfer are suffering from this virus and number of cases increasing rapidly at those regions. In the list of WHO, India hold level 3 and in the category of greater than or equal to 10 case category. This is good for India until now that the number of cases is under controlled. According to the population of India which is more than 135 crores, the community transfer is very easy in this country. But the Government of India declares lockdown and quarantine to all the passengers who came from outside the India. According to Centre for Strategic and International Studies [15], the effect of COVID-19 on global economy also started from China. China was the first country whose economy badly affected from this virus. Chinese economy falls badly. The automobile sales also reduce 80% globally. China’s export falls down up to 17.2% in January and February 2020. Global GDP at this time also affected from 2.9 to 2.4%. And it predicted that in the year 2020, the global GDP will reduce to zero in worse case. According to United Nations, the foreign direct investment will reduce up to 5 to 15%. The effect of COVID-19 on the internal economy is showing a bad scenario of financial crisis. The tourism and travel sector also falls down due to this virus. Even it is predicted that the global air carrier will get loss between $63 billion and $113 billion. Every business of the world is suffering and international film making is also included in this crisis and loosing market of $5 billion. This epidemic causes restaurant business, sports, and energy sectors also suffer [15].

The different impacts of this epidemic on India are trying to predict. The rate of increase of infected patients shows that the journey of this coronavirus is unstoppable. The condition of India in this lockdown is going to worse economically. India is facing problems in different sectors which lay down the growth of the country. Different forecasting methods are available to predict the impacts of this epidemic on country growth. Artificial neural network (ANN) is a technique that utilizes prediction and forecasting in several domains of science and technology. Because of its ability to make mathematical model in both linear and non-linear domains, ANN predictive technique has been used for several prediction purpose like weather events [16, 17], stock market [18], and cloud classification [19, 20]. Apart of ANN, several other techniques or modelling is used for forecasting such as probabilistic method [21, 22], fuzzy model [23, 24], linguistic model [25], Holt-Winters’ methods [26], exponential smoothing model [27], linear regression method [28], and ARMA model [29].

In this paper, a critical review of impacts on major driving sectors is performed due to COVID-19 in context to India. The main contribution of this paper is listed as follows: first, the focus is on initiation of COVID-19 and origin of coronavirus with the effect on different countries. Second, the effect of coronavirus on different countries and India, economically, financially, and life loss is discussed. In Section 2, classification of source of COVID-19 and major impacts is studied. Impact on major sector that drives any nations is studied and discussed. In Section 3, different mathematical modelling is applied for forecasting with problem formulation. Further, in Section 4, prediction results according to different modeling techniques and future roadmaps come from these epidemic challenges. In Section 5, the major challenges faced by the people of India and government are discussed. Finally, the discussion, recommendations, and conclusions are drawn in Section 5.

## Classification of source of major threats/impacts

As per the above discussion, COVID19 changes the world population’s scenario in three to four months. In India, the number of cases is increasing rapidly. Even after the lockdown, the cases in India don’t fall so far. India’s government assumed that lockdown affects coronavirus spread and it will be under control, but few places in India, the numbers are increasing and not under control. A research article predicts with a mathematical model that the number of infected will be 364 million with 1.56 million deaths [30]. This is a biggest threat in India because population of India is very dense in most of the part of region. The most populated state Uttar Pradesh has more than 1000 infected. Most of the area in Uttar Pradesh is lockdown and seal. Indian government distributed the corona patient region in three zones, i.e. red, orange, and green zones. To understand these zones, it is essential to discuss India’s dense population, where the chances of community spread are much higher. Table 1 illustrates the picture of population of India state wise 24.

**Table 1:** States/UTs wise population of India [31] (estimation of 2019)

S. No.	State name	Population (2019)
1	Uttar Pradesh	233378519
2	Bihar	122256981
3	Maharashtra	121924973
4	West Bengal	98662146
5	Madhya Pradesh	83849671
6	Rajasthan	79584255
7	Tamil Nadu	77177540
8	Karnataka	66834193
9	Gujarat	64801901
10	Andhra Pradesh	53390841
11	Odisha	45861035
12	Telangana	38919054
13	Jharkhand	37933898
14	Kerala	35461849
15	Assam	35080827
16	Punjab	29875481
17	Chhattisgarh	28989789
18	Haryana	27793351
19	Delhi	18498192
20	Jammu Kashmir	13468313
21	Uttarakhand	11140566
22	Himachal Pradesh	7384022
23	Tripura	4112223
24	Meghalaya	3320226
25	Manipur	3048861
26	Nagaland	2218634
27	Goa	1564349
28	Arunachal Pradesh	1548776
29	Puducherry	1394026
30	Mizoram	1222134
31	Chandigarh	1142479
32	Sikkim	680721
33	A and N Islands	411278
34	Dadra and Nagar Haveli	384058
35	Ladaakh	279924
36	Daman and Diu	223165
37	Lakshadweep	72172
	Total	1353890423

**Fig. 4:**
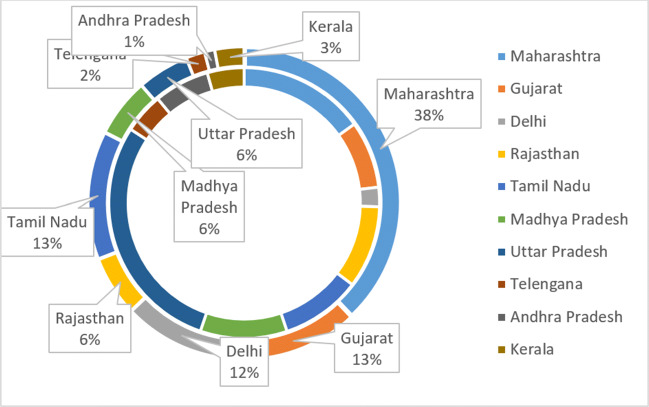
Infected people in top ten populated states in India

It is clear from Table 1, that Uttar Pradesh, Bihar, and Maharashtra are the most populated states of India. These states are on most critical condition of coronavirus spread. The government is trying to avoid these states through complete lockdown but with the time, government fails to stop this virus. Figure 4. shows the population of top 10 states with maximum infection.

**Fig. 5:**
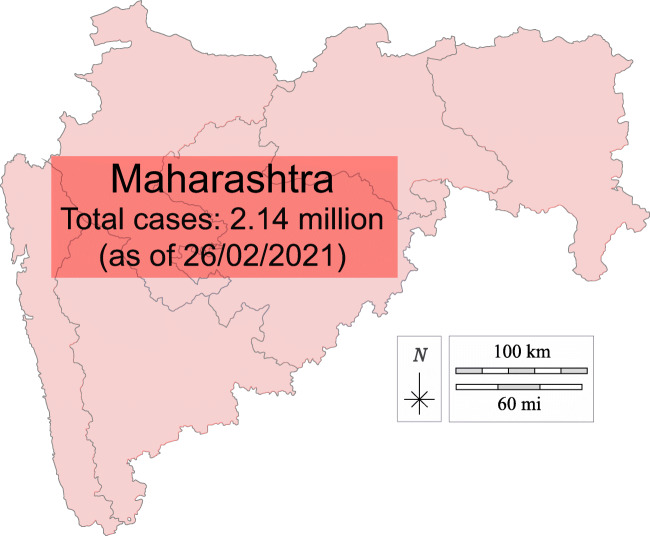
Corona threats in Maharashtra state of India. Modified from [33]

Therefore, government decided to put the infected region in three zones and when it detected, the zone will be completely sealed. No any person or any services provided by the other to this zone. Only government staffs will supply the requirement of the zone. The three zones divided are as follows [32]: **Red zone:** Those districts of India, which has substantial number of infected cases, will come under red zone. It illustrates like this, if the number of infected cases everyday was more than 6, then it will count under red zone. **Orange zone:** Those district or area where limited number of infected cases encountered and no such positive cases encountered in few days, will come under orange zone. **Green zone:** The district with no corona-positive cases anymore will come under green zone. As per Table 1, the number of red zone is rapidly increasing in Maharashtra and Delhi. According to the population in few areas of these states, government declares complete seal to those areas. Figure 5 shows the condition of Maharashtra state in India which is divided in different zones [33].

**Fig. 6:**
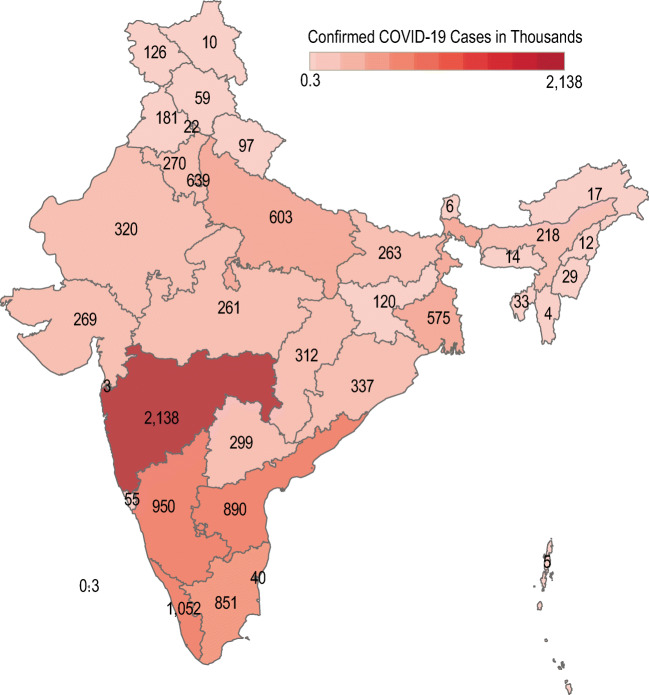
COVID-19 threats map of India. Modified from [33]

A large region of Maharashtra comes in the red zone, as shown in Fig. 5. The probability of community spread in Maharashtra is very high. Figure 6 shows the COVID-19 spread in India [33]. Except Maharashtra, Rajasthan, Gujrat, Madhya Pradesh, and Gujrat are the states which encountered 1000 to 4000 infected. Government of India is taking strict steps in these states. The area which encountered any case of coronavirus, the government seal those areas completely and cut all type of social communications from outside the area. This action really works and reduces the patient in the area encountered.

**Fig. 7:**
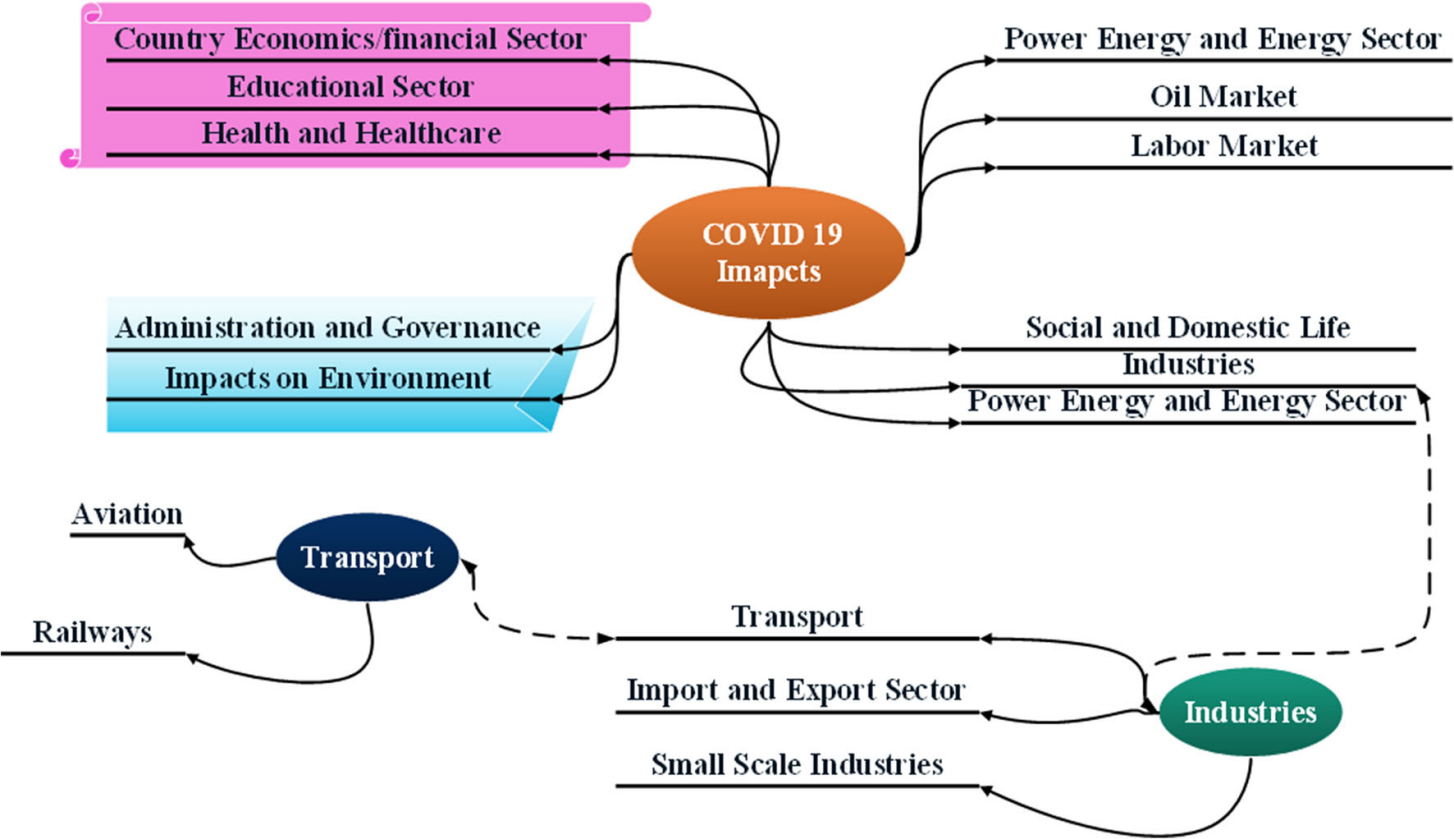
Detailed classification of chart of major impacts (considered in this study)

Due to this COVID-19 attack in India, every sectors of this country like financial/economics sectors, education, health and healthcare, environment, social and domestic industries (import and export, retail market, transport and travel (aviation, public, railways, metros, and private), small industries, hotels), power and energy, oil market, labour/daily wedge market, entertainment/film industry, jobs and employment, administration, governance and politics, environment, and social life are badly affected. The next sub-sections are discussed impact analysis on aforementioned sectors. To ease of understanding, a detailed classification of the chart of major impacts considered in this study is given in Fig. 7.

### Impacts on country’s economics/financial sector

**Fig. 8:**
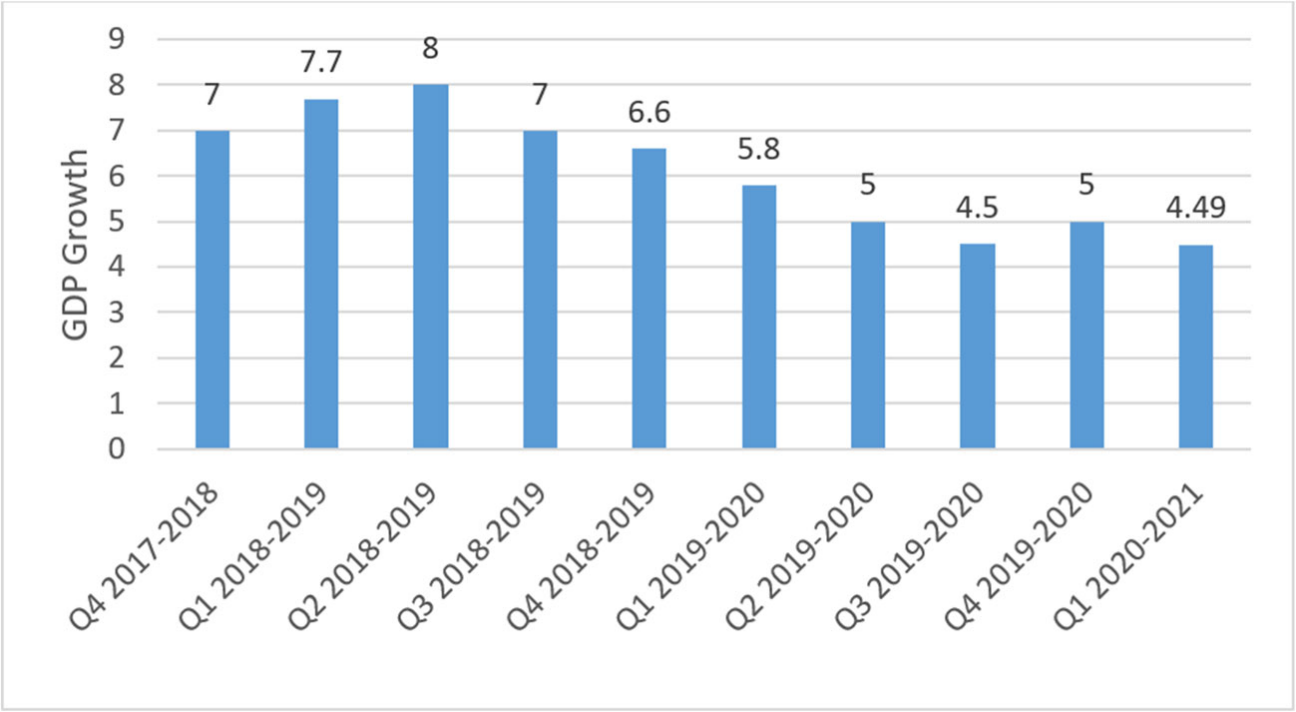
GDP rate of last 5 years of Indian economics

The GDP of India from last 6 years is on lowest in the third and fourth quarter of this financial year 2019–2020. The only region is spread of COVID-19. The GDP of India in the last quarter fall down to 4.8%. This value of GDP is the least value in last 6 years. Even at the time of the global economic crisis in 2007–2009, India’s GDP was much better than today. Hence, it proves that COVID-19 impacted a lot on Indian economy. It is estimated in a report of Klynveld Peat Marwick Goerdeler International Cooperative (KPMG) that the second half of the 2020 will be better than first half and Indian GDP will grow again with growth of 5.3 to 5.7%. Figure 8 shows the GDP rate of India in last 5 financial years [34].

### Impacts on educational sector

COVID19 pandemic also significantly affects the education sector; India’s education sector is valued at USD 101.1 billion, and India’s government spend INR993 billion in the education sector and INR30 billion in the skill development sector. Skill institutes in India provide training to the rural and urban labour class more than 10 million annually, but due to India’s lockdown, the skilled manpower decreases to 10–15% [34]. This will increase unemployment in India in only one and half month. So many examinations and degree distributions are put on hold due to this lockdown period. In the academic year ending, schools, colleges, and universities face closure, and due to which the examination of students is also put on hold. In total, 39,931 colleges and 993 universities are in India and every colleges and universities are facing closure. Few universities and colleges start to educate students through online mode to reduce the students’ losses in this academic year 2020–2021. But it is very tough for the schools, colleges, and universities to cover the contents during lockdown. Due to COVID-19 outbreak, the admissions in the school, college, and universities also going to be impacted. The session of the students get delay and it impacts student’s admission a lot. This is also a financial loss of the country with education loss and skill loss. This may be increase cause of unemployment in the country.

### Impacts on health and healthcare sector

**Fig. 9:**
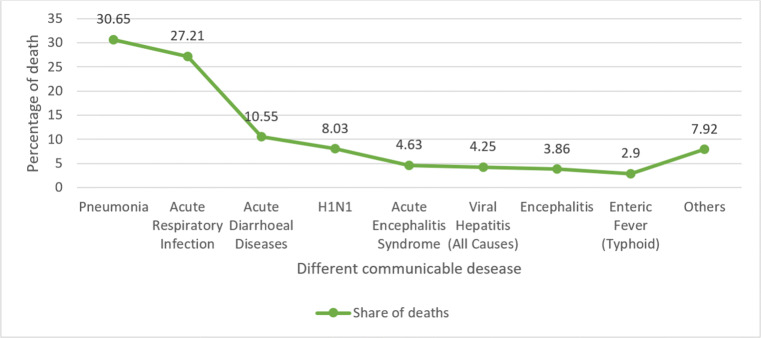
Death in 2018 due to different communicable diseases in past year

Health and healthcare sector should improve in developing countries like India. But due to lockdown and COVID outbreak developing more precisely realized their healthcare facility and infrastructure whether it sufficient to handle this pandemic situation or not? And it’s also one of the major reasons countries like India do not have well-planned healthcare facilities, improving their health sector very rapidly to fight this situation and succeeding from village level to city level. Due to India’s lack of health and healthcare infrastructure, so many deaths were encountered due to different types of the disease annually. Figure 9 illustrate the death due to communicable disease in India in the year 2010.

**Fig. 10:**
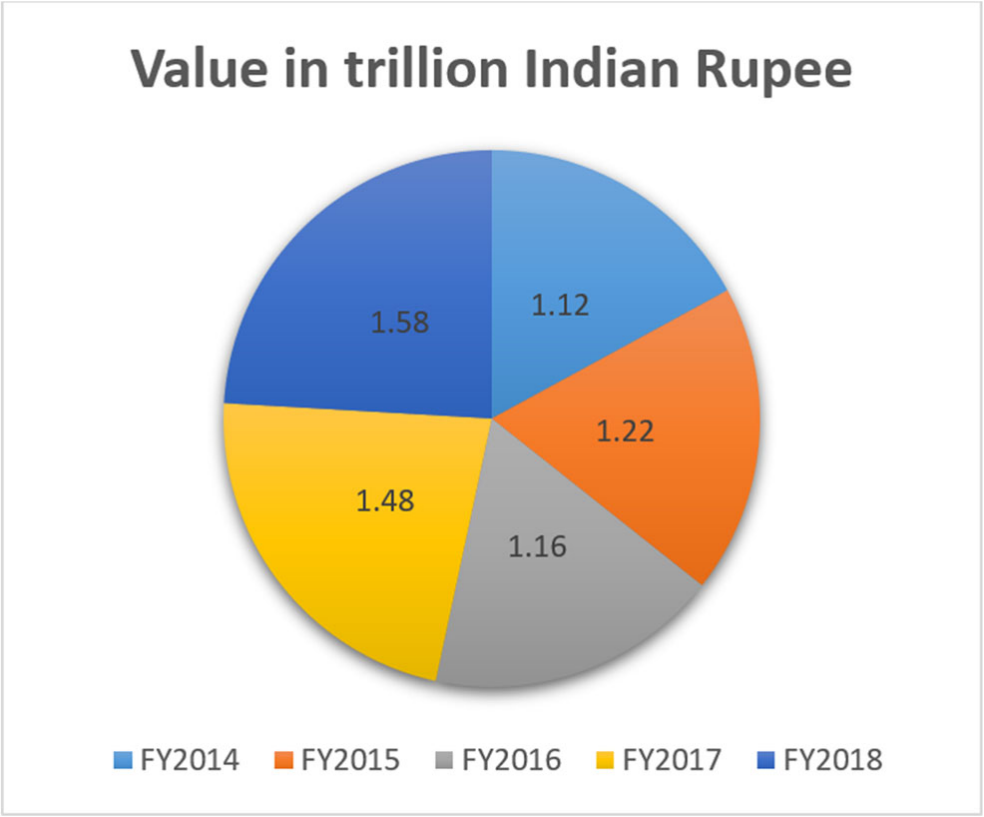
Total expenditure on health and healthcare sector by Indian government from FY 2014 to 2018

In 2019, Global Health Security Index measured country preparedness between a score of 10 to 100 for this type of pandemic. India is on the rank of 57 out of 195 countries which is very lower than Italy and China. The government of India spent only 1.28% of its GDP in 2017–2018 and it was 1.02% in 2016–2017. If it is compared from 2009 to 2010, then it is estimated that per capital expenditure on health and healthcare is more than twice from 621 per person to Rs. 1657 per person in 2017–2018 [35]. But it is estimated by few social and health care organizations that it is still lower than other countries. The USA spent most of its GDP, approximately 18%. Therefore, Indian government needs to invest more on health and healthcare sector. The current Government of India estimates 2.5% expenditure of GDP by 2025 in this sector. Figure 10 illustrates the total expenditure of the Indian government in the health and healthcare sector until 2018 [36].

### Impacts on social and domestic life

With huge population, India holds a mass quantity of labours and farmers, who earn every day and spent every day on food and lodging facility. The social value in India understands with the quote “Unity in diversity.” The people of India like to share their problems with each other. It is most common in this country that someone find to help others in every manner like financially and personally/ socially. Due to COVID-19, government announced to maintain social/physical distancing to avoid spread of this virus. The social distancing means no work and no food for those who earn every day and spent every day. Table 2 illustrates the income level categorization of Indian population [37].

**Table 2:** Annual income wise categorization of Indian population (in INR)

Income quintiles	Income range
Poorest quintile (poorest 20%)	1000–33,000
2nd quintile	33,001–55,640
3rd quintile	55,641–88,820
4th quintile	88,821–159,600
Richest quintile (richest 20%)	≥ 159,601

From the data of Table 2, it is clear that 20% of the population of India are poor who earn every day. And the annual income of this category started from INR 1000. if consider INR 33,000 salary annually, then it is approximately INR 2750 only which is less than INR 100 per day. In this lockdown time, these workers are suffering from lack of money and food. The social distancing announces by the government strictly applicable due to which these people are helpless and dependent to the other class of community who can distribute food to them. In most of India’s cities, government and social activists are distributing food to those category people. Government announces to deposit some amount monthly in their accounts to survive in this lockdown time. On the other hand, in these difficult times of COVID-19, ambiguity, and lockdown, many may not be immune to psychological stress. Thus, to cope with anxiety and unease during the COVID-19 outbreak, India’s government introduced various helpline numbers for professional assistance on psycho-social problems among the unpredicted lockdown population.

### Impacts on industrial sector

**Fig. 11:**
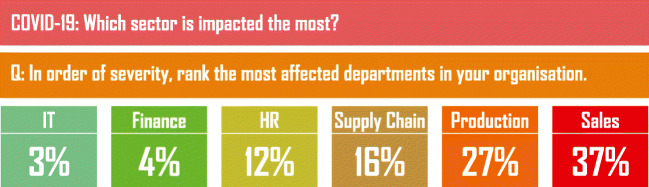
Impact on different parts of industries in India due to COVID-19 and lockdown. Modified from [38]

An article in ‘cio.com’ estimates the total loss in industries’ growth in this lockdown period. The drives and jobs impacted globally but in India, the unemployment was already on large scale, and the government announces this lockdown. The economy and industrialization stop in India in the past month. Figure 11 generated by ‘cio.com’ illustrate some industry categories that impacted a lot in this lockdown [38].

The sales sector in industry completely affected because people are not going anywhere in the market/multiplexes for shopping, and all the online e-commerce platform that gained the largest growth in past years in India are closed now. Due to maintaining social distancing, the sales of the industry stop and all the companies are facing financial loss and closure. Production sector also impacted a lot due to closure. IT sector is little bit stable because employees of this sector are working from home on their projects/tasks. Finance sector also less impacted because banking and financial activities are running in the country by considering the essential services.

#### Import and export sector

As discussed above, China export and import affected in the month of January which reduce the global development. China is one of the large exporters in the world. India also holds a position in export import. According to trading economics, Indian export income falls down to 34.57% from previous year which is approximately USD 21.41 billion. It is most lower percentage after 2016 [39].

**Fig. 12:**
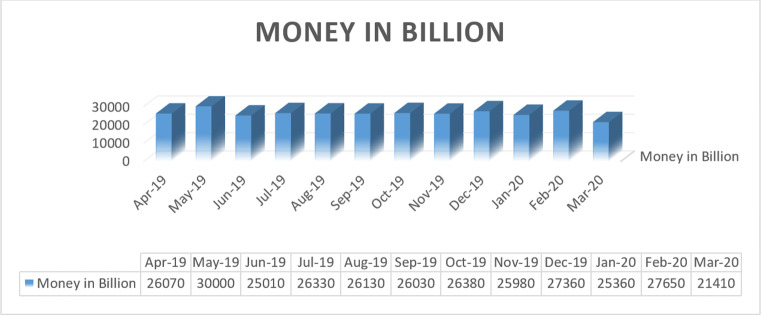
Export income of India from April 2019 to March 2020

It is clear from the Fig. 12 that the export income of India reduces INR 21,410 billion which is lesser than December month. This deduction in export impacts on the economy of India very badly. Table 3 illustrates the commodities which India export.

**Table 3:** Commodities export by India

Commodities	In months Feb and March	Previous	INR
Non-basmati rice	114788.1	102165.1	Million
gems and jewellery—total	21275.65	21146.59	TML
Non-basmati rice	12650.78	11883.3	Million
Gems and jewellery—cut and polished diamond	9897.14	11757.08	TML
Gems and jewellery—gold jewellery	6495.97	4421.1	TML
Gems and jewellery—silver jewellery	1688.68	1443.36	TML
Manufactured goods	1359.59	1392.75	Billion
Gems and jewellery—rough diamonds	546.92	530.17	TML
Engineered goods	517.91	445.17	Billion
Chemical and products	280.13	286.53	Billion
Gems and jewellery—gold medallions and Co	241.8	515.97	TML
Agricultural products	211.5	233.8	Billion
Gems and jewellery—coloured gemstones	124.06	302.55	TML
Textile Excl. Ready-made garments	109.81	107.89	Billion
Electronic goods	83.81	72.9	Billion
Ores and minerals	29.52	25.87	Billion
Leather and products	27.27	29.01	Billion
Handicrafts Excl. Hand-made carpets	11.31	10.64	Billion
Gems and jewellery—pearls	0.71	1.38	TML
Gems and jewellery—synthetic stones	0.55	0.36	TML

It is observed from Table 3 that, except for rice, the export of other commodities falls. Rice is a food item and in the time of COVID19, most of the countries are suffering from this virus and announces a lockdown. This situation requires food items in a large quantity. Therefore, the export of rice is increase but the remaining commodities export decrease drastically. Similarly, the import of India reduces to 28.72 percent from August 2016. It is also low value in the last few years. To see the danger level of COVID 19 in India and worldwide, the Indian government stop importing goods from outside the country. In May 2019, India import cost approximately INR45,350 million, which reduces up to INR31,160 million in March 2020 [39]. COVID19 spread worldwide and is spreading from human to human touch and from commodities touched by an infected person. To see this, Government of India restrict all import from other countries.

#### Transport

COVID-19 stop to the world and India, after lockdown also stop. Every type of transport in India restricts until the lockdown continues. The population of India travel a lot to reach their offices and also to other cities. To see the human-human spread of this coronavirus, government decided to suspend all type of transport services. Transport is one of the main income sources of Indian GDP. Aviation and railway services are suspended. If the loss in these sectors calculated, then it costs approximately US$18 billion [34].

1. Aviation industry : 464 airports are in India in which 125 airports are under government sector and handle by airport authority of India. These 125 airports of airport authority of India manage 78% of domestic passenger every year and 22% of international passenger traffic. Aviation sector contributes approximately INR 72 billion in the growth of Indian GDP. This revenue goes down to 44% in the starting of 2020. Due to this lockdown, it is estimated that the revenue from aviation industry goes down to more than 70% [40].
2. Indian railway: Indian railway is the third largest rail network in the world. Indian railway completely owned by Government of India and railway has 13,452 passenger trains and 9141 freight trains. India invests a large budget from its GDP on India railways. Up to November 2019, the total revenue of Indian railway was US$16.34 billion. But due to complete lockdown announces, Indian railways also stop its passenger’s service from last week of Feb 2020, and only good and essential item supplies permitted by good trains and this will continue until the lockdown endures. A large amount of income from Indian railway suffers in these lockdown periods [41].
3. Public and private transport: Public and private transportation sector has been one of the key sufferers of COVID-19. From rickshaw pullers to public, private, and metro trains, all have been pretentious frugally by the pandemic. India’s inclusive energy demand fell by 11% in March 2020. Because of lockdown in numerous countries, the demand for passenger transport has been undesirably hit. The cargo segment has had a varied short-term consequence in terms of conveyance demand. There is a swell in demand for truck drivers in transportation of indispensable goods. For example, there is 40 to 60% growth of product being moved into grocery stores and warehouses in USA since COVID-19 blowout happening. Though the supply chain disturbance and stoppage caused by COVID-19 is predictable to demolish freight demand in the medium-term. Urban freight sections in India have also had a mixed short-term consequence in terms of transportation demand. Meanwhile, in February, the online food orders have released by 20% while online grocery orders are abundant. Likewise, lockdown does not let to use even the private transport deprived of foremost emergency condition and thus huge work and economic loss [34].

#### Small-scale industries

A large amount of the Indian population depends upon small-scale industries. All the industries shut down due to lockdown. The workers and employees get back to their homes for a safe staying. Small-scale industries cannot pay those employees in the shutdown because those companies’ production completely shut down. The government of India has announced different types of schemes for these types of industries like rebate in bank interest, rebate on loan installment, and a fixed amount of income to those unemployed due to this lockdown and work in small scale industries [42].

### Impacts on oil market

**Fig. 13:**
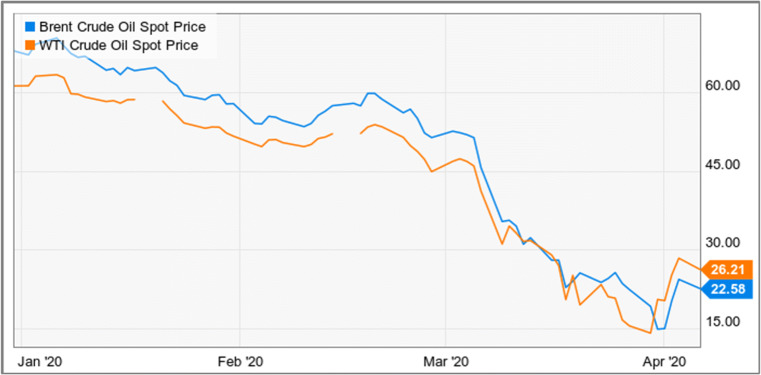
Brent and WTI Crude Oil Spot Price fall down globally [43]

The oil market is also one of the sources of income for the Government of India. Transport and different industries run through the oil market. This lockdown due to COVID-19 banishes the oil market. Also, internationally, the oil market impacted due to coronavirus. The main exporter of oil like gulf countries, America are suffering from this coronavirus. Globally the oil market crashes due to which Indian oil market also crashes. India imports more than 80% of oil consumption in India. Indian oil companies, particularly the E&P space (upstream) like ONGC and Oil India, may aspect harsh times in the future as of bigger burden to retail their products at lesser prices ahead. The refiners and distributors (downstream) like HPCL, Reliance, and IOCL are expected to see better margins in the upcoming quarters, once the demand gears up once more. Because for the stowage, if the Indian companies can be able to their stockpile well, this is an upright time to purchase and backup oil for upcoming use. The global oil prices are varying rapidly which are effecting Indian economy. As on 24 April 2020, the oil market variation is shown in Fig. 13 [36]. It can be examined from this figure that the crude oil price is going in less which happens first time in oil market due to lack of closure facilities, storage, less demand, and huge economic loss of stopping of production of oil wells globally.

### Impacts on labour market

As discussed in the previous sub-section, more than 20% of the Indian population are in the poor category, and they earn every day and spent every day. When the lockdown started in India, these 20% population of India’s people lost their jobs, which was a significant burden on the Indian economy and the government. Labors are gone to their native places to survive in the lockdown. Only a few laborers stay in the city area of the country. Due to the shortage of labor, so many sectors impacted like construction, transport, etc. India’s government took the initiative to provide them a survival life by paying them a few amounts in their bank accounts. Still, the industry sectors survive lack of labors [34].

### Impacts on power and energy sector

**Fig. 14:**
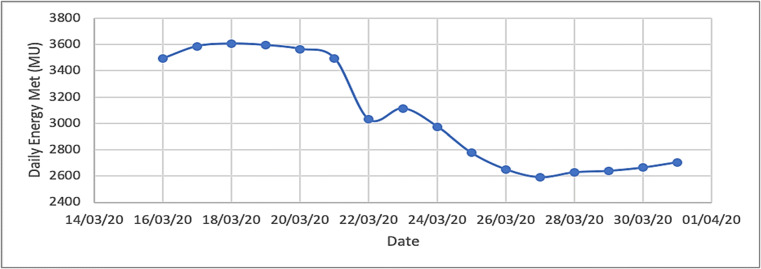
Energy demand (MU) between 25 March and 31 March 2020 of India [45]

The total influence of the COVID-19 health disaster on the worldwide energy sector has seen oil prices and demand drip dramatically, but it may have exposed the door for prospect in the renewable energy sector. Earlier month of April 2020, the oil and gas sector was already sense a negative impact on its processes, felt slightly due to increased emphasis on the significance of climate changes. Power technology affects the major stakeholders of power on it’s generation and supply. The lockdown has instigated electricity demand to drop as industrial electricity consumption grasps a maximum portion in most countries’ consumption mix. Coal-based generation holds a higher than 40% share in the global electricity generation. The reduction in electricity demand has enforced a number of coal plants to operate at lesser capacity, sinking the overall generation mix [44]. Likewise, some nuclear facilities have worked slightly at lesser capacity due to remote operations. The COVID-19 lockdown has ran to blackout of all but essential commercial deeds throughout the country. Nearly, 1.3 billion residents are appreciative to persist within the boundaries of their homes and, only, allowed to work from home. Subsequently, the electricity demand from industrial and commercial consumers has decreased ominously, while the residential demand is likely to have raised. As said by the Power System Operation Corporation (POSOCO) of India, the energy demand met on March 16, 2020, which can be deliberated as a business-as-usual scenario, was 3494 MU as paralleled to 3113 MU on March 23, 2020, a day of voluntary curfew. It lesser to a range between 2600 and 2800 MU between March 25 and March 31, 2020. This drift is showed in Fig. 14 [45].

Following may be the major impacts of COVID-19 on Indian power market:

1. Firstly, a key risk from the COVID-19 pandemic on the loss of incomes due to lessening of huge demand (industrial and commercial) of by now the fraught distribution companies (DISCOS) as well as their inability to cope up the cross-subsidies in case to the less-tariff paying customers.
2. Secondly, the utilities would also have already day-ahead or long-term power purchase agreements/ commitments with the generating companies (GENCOS). The true evaluation and degree of this risk would only be identified once a quantitative analysis is directed.
3. Thirdly, at operational level, DISCOS would have to comprise deviance in demand and supply configurations at a sequential and locational level.
4. Finally, during this retro, serious infrastructure such as electricity grid would have to be run with least personnel.

**Fig. 15:**
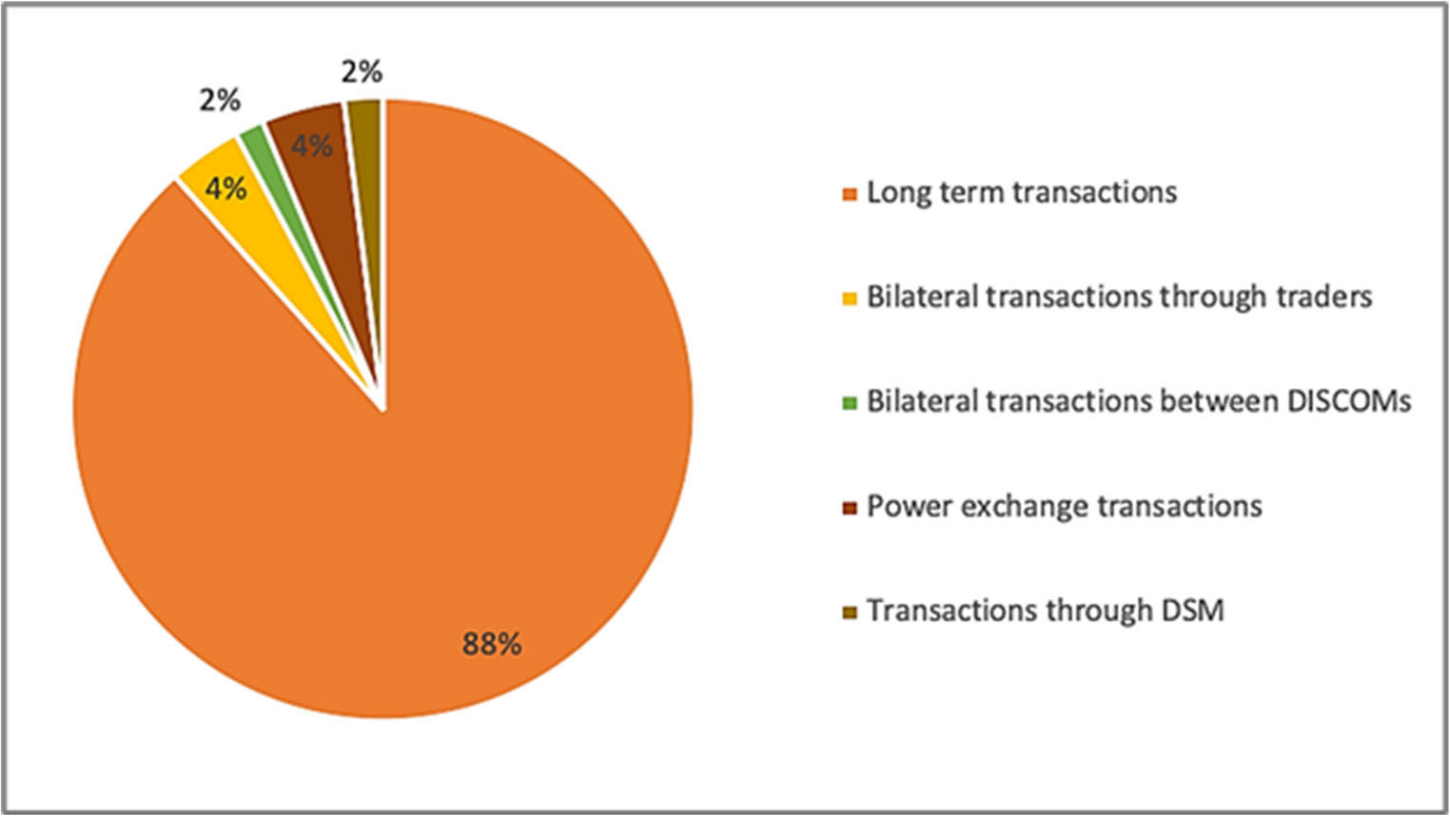
Share of market sections in total electricity generation 2018–2019

As seen in Fig. 15, the trade of wholesale power market comprises just 4.3% of the total electricity transactions. Though, the transactions over the power exchanges have grownup over the last decade. The Indian Energy Exchange (IEX) has seen an evolution from 2616 MU in FY 2009 to 52,241 in FY 2019 [46].

Another impact of the COVID-19 pandemic on the power markets is in relations of the market dynamic. It can be detected that there is a hollow in the clearing volume and the market-clearing price, which accords with the progressively cumulative shutdown actions taken by the government as a reaction to COVID-19. Thus, presently, Indian power sector at an initial phase of conversation on the effects of COVID-19. The long-term impacts of the existing state of affairs would only turn out to be outward with time.

### Impacts on environment

A global COVID-19 pandemic that is suing people survives surely should not be seen as a way of carrying about environmental alteration either one. The month of May, which generally accounts peak carbon emissions due to the disintegration of leaves, has noted the lowermost intensities of impurities in the air after 2008 financial disaster. The first thing to consider, says Kimberly Nicholas (sustainability researcher, Lund University, Sweden), is the dissimilar causes that emissions have fallen. Take transport, e.g. which marks up 23% of worldwide carbon emissions. The emissions have dropped in the short term in nations wherever public health processes, such as keeping people in quarantine, have cut needless travel. Pouring and aviation are crucial providers to emissions from transport, paying 72% and 11% of the transport sector’s greenhouse gas emissions (GHGs), respectively [47]. As stated by Cleveland, OH [48], following temporal environmental impacts can be seen:

1. The temporary restriction of air and major transport travel could lead to cleaner air.
2. The quarantine/lockdown time could cause additional use of single-use plastics, which could discover their way into water bodies.
3. An advantage of preventive travelling is devoting more time reconsidering how we use positive energy.

**Fig. 16:**
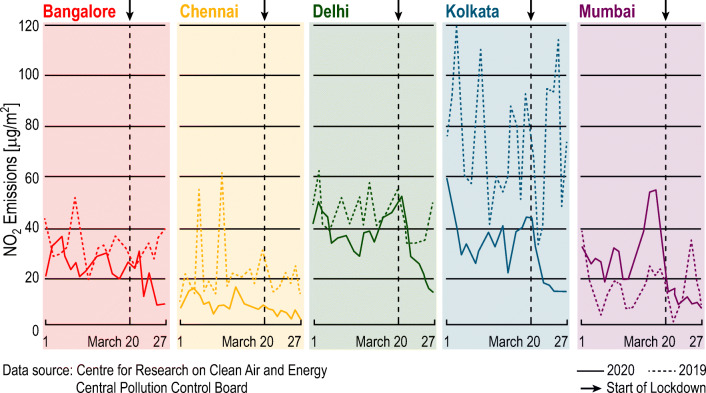
The average NO2 emission data of five metros cities of India during lockdown. Modified from [51]

The difference in air quality index over the world from January to March 2020 can be seen from Earther website [49] through an interactive map that shows how air pollution has improved over COVID-19 outbreak. In India, the results are alike too; March 22, 2020, was the “1Janata Curfew” (voluntary curfew), subsequent which, a noteworthy incline in air pollution levels was observed across the nation. Many of the big cities such as Delhi, Bengaluru, Kolkata, and Lucknow regain their average Air Quality Index (AQI) keep on within only two digits. While the whole closure of India’s economy was intended to break the blowout of COVID-19, it is having an ancillary wellbeing benefit of clear and fresh air that millions of individuals were unpleasant. As vehicles/major transport stay off the road, construction activities are put on hold, and plants/industries stop production, the levels of microscopic particulate matter (PM) 2.5 starts to down. There was a remarkable enhancement in air quality in the Nation Capital Region (NCR), as the injurious PM10 and PM2.5 levels were lower down by 35–40% in Delhi as it was in 2018 or 2019 [50]. The average NO2 emission (micro grams per sq. mt.) data from the Central Pollution Control Board (CPCB), part of India’s Environment Ministry, was collated by the Centre for Research on Energy and Clean Air (CREA) shown in Fig. 16 of five major metros cities of India. It clearly may observable that the fall down of NO2 level in each city has never been recorded before [51].

The water bodies have also been cleaner, and the rivers Yamuna and Ganga have realized substantial upgrading since the nationwide lockdown. As per the real-time water monitoring data of the Central Pollution Control Board [51], the average water quality of 27 points of the Ganga realized, apposite for bathing and propagation of wildlife and fisheries. Hence, COVID 19 has made its positive impacts on the environmental point of view. Still, once regularity is renovated, it would be undeniably essential to make sure that all can maintain these improved levels of environmental measure up to an extent. Furthermore, summarizing India’s different sectors’ aforementioned significant impacts, it needs to take sufficient time and well strategic planning in all front by the government and policymakers so that country’ economics and individual life and interest secure in this most challenging time.

## Mathematical modelling and problem formulation

In this work, Holt-Winter Model [26], Exponential smoothing [27], Linear Regression [28], Seasonality Linear Regression [28] are used to predict and forecast the results of impacts consider due to COVID19. After performing forecasting through these models, the results are compared, and we conclude the best models or similarities. Linear regression model and Seasonality Linear regression model approximately show the same performance, but a seasonality matrix is evaluated in Seasonality linear regression. Whereas, in direct Linear Regression, no need to evaluate the seasonality index. The proposed model formulations are applied to different sectors, i.e., COVID patient rate, daily cases encountered, number of deaths encountered, GDP of India, and unemployment. These problems directly link and affect a country’s economics and finance position, due to which the development of a country like India pushback to 10 years. Different types of problems are considered in different models, but the final results are evaluated for every problem through every model.

### Exponential smoothing model

Exponential smoothing is a popular modelling method to forecast univariate time series data. This method assigns weightage to all past observations in an exponential decreasing pattern as the observation gets older. It means, the recent observations will assign higher weightage than older observations. The mathematical modelling of this exponential smoothing model is:

1 $$ F_{t+1}=\alpha D_{t}+\left( 1+\alpha \right)X_{o} $$

where *F* is forecast, *D* is demand, *α* is the smoothing constant $\left (0<\alpha <1\right )$, *X*_*o*_ is the first fitted value at time 1, and *t* is the total observation with time ∈ positive numbers.

For formulation of problem, the daily encountered cases of corona disease are considered, which is taken from April 1, 2020, to May 14, 2020. This time series data is in approximately increasing order.

2 $$ \begin{array}{@{}rcl@{}} Cde = [146, 601, 545, 516, 529, 701, 489, 573, 565, 809,&& \\ 875, 846, 759, 1248, 1034, 883, 1060, 922, 2013, 1250,&& \\ 924, 1541, 1290, 1669, 1408, 1836, 1607, 1561, 1873, &&\\ 1738, 1801, 2394, 2442, 2806, 3932, 2963, 3587, 3364, &&\\ 3344, 3113, 4353, 3607, 3524, 3763, 3942]&& \end{array} $$

3 $$  Cde\ =\ \left[D0,\ D1,\ D2,\ D3,\ D4,\ {\dots} ,\ Dn\right] $$

where *n* ∈ positive numbers, and as per (2), the value of *n* (i.e. the number of observations) is 44. Hence, with reference of (1), the 45th value of this time series will be:

4 $$  F_{45}=\ \alpha D_{44}+\left( 1-\alpha \right)F_{44} $$

Similarly, $F_{44}=\ \alpha D_{43}+\left (1-\alpha \right )F_{43}$, $F_{43}=\ \alpha D_{42}+\left (1-\alpha \right )F_{42}$, … … … $F_{2}=\ \alpha D_{1}+\left (1-\alpha \right )F_{1}$.

Therefore,

5 $$  F_{t+1}=\ \sum\limits_{j=0}^{t-1}{\alpha {\left( 1-\alpha \right)}^{j}D_{t}+{\left( 1-\alpha \right)}^{t}F(1)} $$

$$F_{45}=\sum\limits_{j=0}^{44}{0.2{\left( 1-0.2\right)}^{j}D_{t}+{\left( 1-0.2\right)}^{t}\times 620}$$

Let assume *F*_1_ is the average of first ten observations which is equal to 547.4 and assume *α* = 0.2. Therefore, $F_{2}=\ 0.2\times 146+\left (1-0.2\right )\times 547.4=467.12$

$$F_{3}=\ 0.2\times 601+\left( 1-0.2\right)\times 467.12 = 493.896$$

Similarly, for *F*_4_, *F*_5_, *F*_6_, …, *F*_45_ and so on will be calculated. *F*_45_ = 3455.0 which is the range of the data given in (2).

The value of smoothing constant is considered small because the weightage to all the observation was controlled by this smoothing constant. If the value will be small, it will assign weightage to all observation more conveniently.

### Linear regression model

Linear regression is also a very popular method to predict future values on the basis of past values. In this method, a linear line is plotted with the data set line using least square method and trying to minimize the error between trending dataset line and linear line. The distance between dataset point on dataset line and linear plotted line is called error. This linear regression model minimizes the error between these points and give future values. For the same problem which consider in (2), apply linear regression model to find the instance on 45th position. The mathematical equation which implement in this model is as follows:

6 $$  e_{t}=y_{t}-a-bt $$

7 $$  \min\sum{{e^{2}_{t}}}=\sum{{\left( y_{t}-a-bt\right)}^{2}} $$

Differentiate (7) with respect to *t* and with respect to *b* to minimize the error between trending line and linear line plotted. The equation after differentiation of (7) two equations we get, i.e. (8) and (9):

8 $$  \sum{y}=na+b\sum{t} $$

9 $$  \sum{yt}=a\sum{t}+b\sum{t^{2}} $$

where *e* is the error between points on dataset and linear plotted line, *a* is the value of *y*-axis for *t* = 0, *b* is the slop of line, *n* is the number of dataset ponits, *y* is a datapoint, and *t* is time instance.

To find the values of *a* and *b*, the problem of time series in (2) is to be evaluated.

$$\sum{y}=\sum{C_{de}}=80746, \ \sum{t}=1035,$$

$$ \sum{yt}=2499884,\ \sum{t^{2}}=31395,\ n=45$$

By putting these values in (8) and (9), the value of *a* and *b* can be evaluated.

10 $$  80746 = 45a+1035b $$

11 $$  2499884 = 1035a+31395b $$

From (10) to (11), the value of *a* = − 153.2990 and *b* = 84.6806.

Therefore, forecasted value of the time series given in (2) for 45th instance according to Linear regression model is:

12 $$  F_{t}=a+bt $$

$$F_{45}=\ -153.2990\ +84.6806\times 45 = 3657.3$$

### Holt’s model

Holt’s model is also known as the linear exponential smoothing model, which is very popular for trending type data forecasting. This model has included three equations in which the first equation smoothed the value for the last period’s trend, the second equation update the trend over time. The trend is expressed as the difference between the last two smoothed values, and the third equation is used to generate the forecasting results with the help of the first two equations. This method is also called double exponential smoothing. The three equations are as follows:

13 $$  a_{t}=\alpha D_{t}+(1-\alpha )(a_{t-1}+b_{t-1}) $$

14 $$  b_{t}=\beta (a_{t}-a_{t-1})+(1-\beta )b_{t-1} $$

15 $$  F_{t+1}=a_{t}+b_{t} $$

where the slope of the line is given by *b*_*t*_, *a*_*t*_ is the level which represents the smoothed value up to and including the last data, *α* and *β* are the smoothing constant, and *F* represents forecasted results. To prove this model’s forecasted result, the problem that specifies in (2) is again considered and with the help of Holt’s model, 45th instance forecasted value is to be estimated. To solve the problem, some considerations are required with assumptions of the initial parameters:

$$ \begin{array}{@{}rcl@{}} \alpha&=0.2,\ \&\ \beta=0.3\\ a_{1}&=D_{1}=601\\ b_{1}&=\frac{D_{44}-D_{1}}{t-1}=77.69 \end{array} $$

Therefore, from (14) to (15), values of *a*, *b*, and *F* can be calculated as follows:

$$ \begin{array}{@{}rcl@{}} a_{2}&=&\alpha D_{2}+\left( 1-\alpha \right)\left( a_{1}+b_{1}\right)\\ &=&0.2\times 545+\left( 1-0.2\right) \left( 601-77.69\right)\\ &=&527.648\\ b_{2}&=&\beta \left( a_{2}-a_{1}\right)+\left( 1-\beta \right)b_{1}\\ &=&0.3\times \left( 527.648-601\right)+\left( 1-0.3\right)\times 77.69\\ &=&32.377\\ F_{3}&=a_{2}+b_{2}\\ &=&527.648 + 32.377\\ &=&560.026 \end{array} $$

Similarly, for *F*_45_, value will be calculated by Holt’s model using the values of *a*_44_ and *b*_44_.

$$ \begin{array}{@{}rcl@{}} a_{44}&=&4144.4,\\ b_{44}&=&77.96,\\ F_{45}&=&a_{44}+a_{44}\\ &=&4222.3 \end{array} $$

### Winters’ model

Holts’ model used two equations, but winters’ model specifies three equations, first for level component, second for trend component, and third for the seasonal component. This method updates its components at each specified period. The initial values of level and trend are obtained from linear regression. This method uses three smoothing constants for level, trend, and seasonality. The mathematical equations of Winters’ model are written below as:

16 $$  a_{t+1}=\alpha \left( \frac{D_{t+1}}{C_{t+1}}\right)+(1-\alpha )(a_{t}+b_{t}) $$

17 $$  b_{t+1}=\beta (a_{t+1}-a_{t})(1-\beta )b_{t} $$

18 $$  C_{t+p+1}=\gamma \left( \frac{D_{t+1}}{a_{t+1}}\right)+(1-\gamma )C_{t+1} $$

19 $$  F_{t+1}=(a_{t}+b_{t})C_{t+1} $$

where (16) represents level components, (17) represents trend components, and (18) represents seasonality components. To evaluate and verify this model, the problem specifies in (2) is considered again and evaluates the value of 45th instance. To calculate these values, some initial parameters are assumed.

$$ \begin{array}{@{}rcl@{}} C_{1}&=&\frac{146}{2337}=0.0625,\\ C_{2}&=&\frac{601}{2337}=0.2572,\\ C_{3}&=&\frac{545}{2337}=0.2332,\\ C_{4}&=&\frac{516}{2337}=0.2208,\\ C_{5}&=&\frac{529}{2337}=0.2264,\\ \ \textrm{and,}\\ a_{1}&=&\frac{146}{0.0625}=2336=\frac{D_{t+1}}{C_{t+1}}=a_{t},\\ \textrm{with }\alpha&=&0.2,\ \beta =0.3,\ \gamma =0.25,\ \text{and} \\ b_{1}&=53 \end{array} $$

Putting these values on (16), (17), (18) and (19), it is possible to calculate the value of *F* (e.g. in this case *F*_3_).

$$ \begin{array}{@{}rcl@{}} a_{2}&=&0.2\times 2336+0.8\times \left( 2336 + 53\right)=2378.4\\ b_{2}&=&0.3\times \left( 2378.4-2336\right)+0.7\times 53 = 49.82\\ C_{7}&=&0.25\times \frac{601}{2378.4}+0.75\times 0.2572=0.2561\\ F_{3}&=&\left( 2378.4+49.82\right)\times 0.2332 = 566.26 \end{array} $$

Similarly, the value of 45^*th*^ instance of the problem formulated according to Winters’ model is as mention below:

$$ \begin{array}{@{}rcl@{}} a_{44}&=&16688,\\ b_{44}&=&266.3209,\\ C_{45}&=&0.2425,\ \text{and}\\ F_{45}&=&4111.8 \end{array} $$

**Fig. 17:**
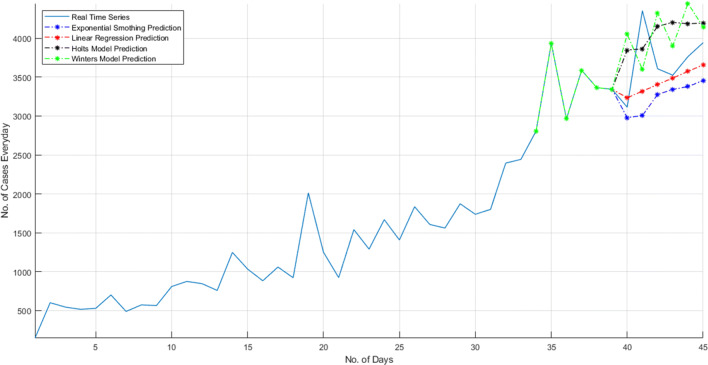
Prediction of daily cases with different models and compared with actual data

The problem considered in (2) is forecasted for its 45^*th*^ instance from four different modelling technique such that exponential smoothing model, linear regression model, Holt’s model, and Winters’ model. The 45th instance of the time series problem is very near to the approximation. The different results from different models are given in Table 4. And Fig. 17 illustrates these prediction as compared to actual data.

**Table 4:** Output table of mathematical models for a problem specifies in (2)

Instance	Real	Exp.	Linear	Holt’s	Winters’
	data	smoothing	regression	model	model
40th	3113	2978.7	3233.9	3847.0	3618.0
41st	4353	3005.6	3318.6	3862.9	4213.9
42nd	3607	3275.0	3403.3	4153.1	3944.7
43rd	3524	3341.4	3488.0	4203.3	4484.5
44th	3763	3378.0	3572.6	4186.0	4138.4
45th	3942	3455.0	3657.3	4222.3	4111.8

As shown in Fig. 17, It is clear that different models give different predictions due to their smoothing constants and weightage system. If the model estimates the error between the modelling, then it can be evaluated that Holt’s and Winter’s model shows less prediction error. But for the different types of time series problems, it may be varying. The red line in Fig. 17 is going to linear after some instance for 40 to 45 days. But, exponential smoothing followed a pattern; also, Holt’s model followed the pattern, and the final result approximately meets the actual result.

## Results and discussion

Based on the data available of various sectors that have been discussed above, a comprehensive potential impact analysis has been performed with the help of interactive graphs, which has been categorized by four mathematical models such that exponential smoothing model, linear regression model, Holt’s model, and Winters’ model. One problem is formulated in the mathematical model which is daily number of infected cases. But here, including it, six more problems are forecasted which is related to the impacts of COVID-19. To prove the prediction with the help of these model, few more problems related to impacts of COVID-19 in India are considered and illustrated in next article. With the help of these mathematical modelling, the prediction of total corona patient until July, daily cases until July, and number of deaths due to COVID-19 until July is to be evaluated. On the other hand, for India, the prediction of GDP until next 8 quarters and unemployment rate until next 8 are also evaluated. These factors affect the growth of a country and without evaluating these factors, someone cannot estimate that, where it should be lie in next 2 to 3 years.

**Fig. 18:**
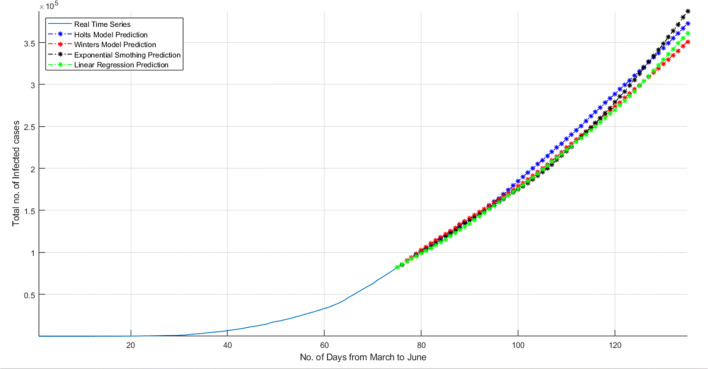
Total infected cases until 14 May 2020 and prediction for next 2 months in India

As shown in Fig. 18, the total number of cases is increasing by time in India. Government announces lockdown until 17 May 2020 and after this date, the lockdown will be open conditionally. But the increasing graph illustrates that the number of cases will increase. Four different mathematical models are showing increasing graph of infected people and will reach to 4 lacs in upcoming two months.

**Fig. 19:**
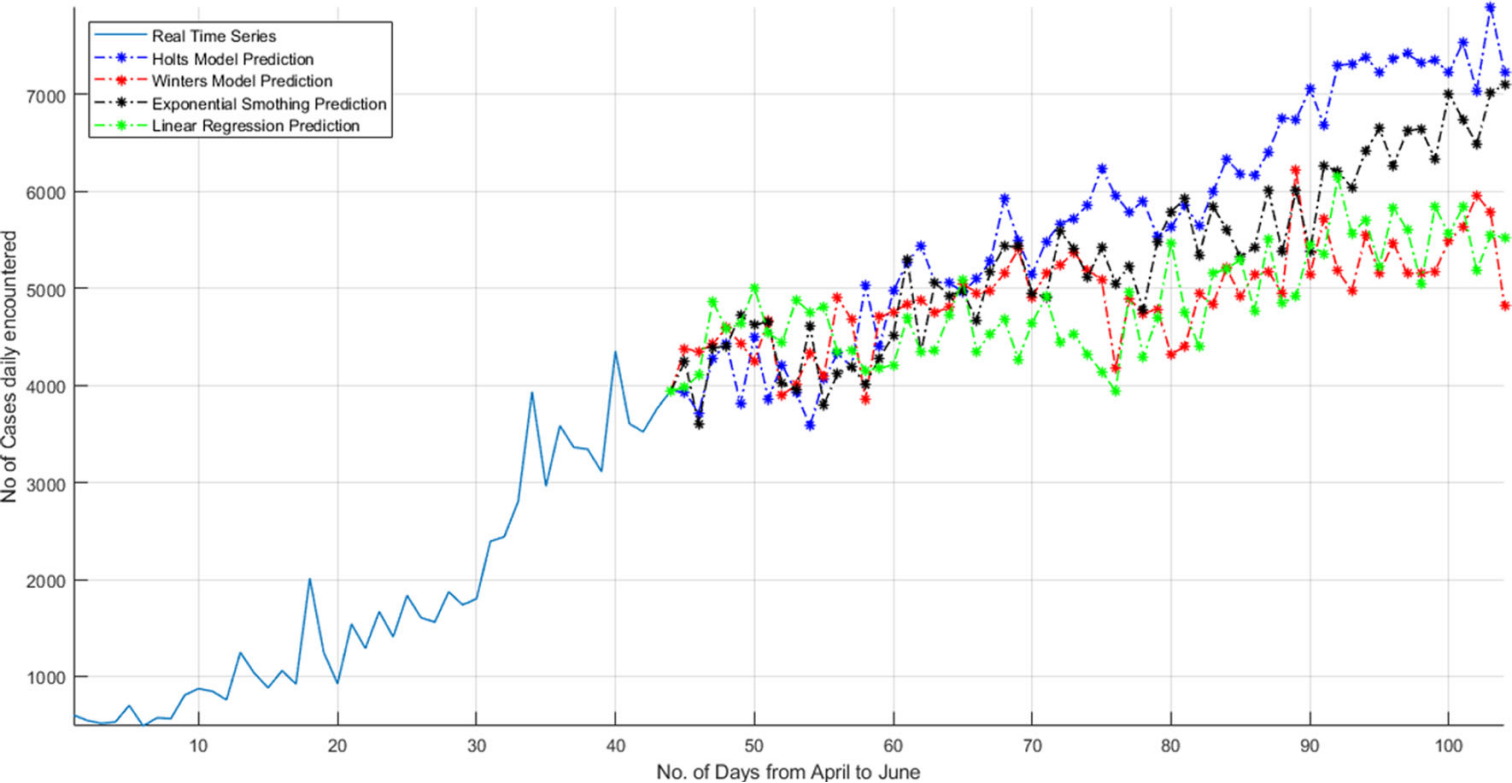
Total daily cases encountered until 14 May 2020 and prediction of every day cases encountered in India for next 2 months

As shown in Fig. 19, the daily cases encountered in India are started from 3 and later on it reaches to 600. But in recent few days, the daily encountered cases reach to 3500. The prediction from four different model illustrates that the daily encountered cases will increase with time and this epidemic will cover a huge population of India.

**Fig. 20:**
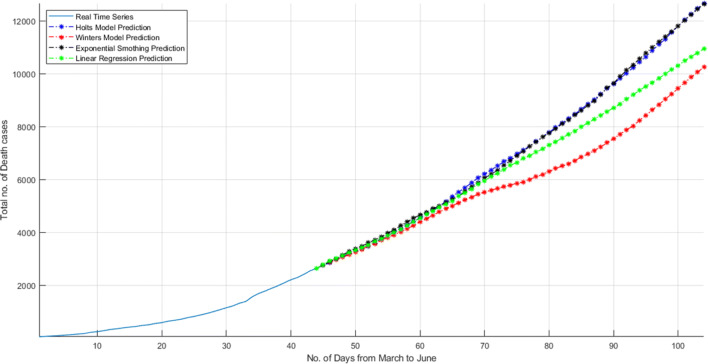
Total deaths encountered until 14 May 2020 and prediction of death encountered in India for next 2 months

Until now, the number of deaths is under 3000. But as shown in Fig. 18, the number of infected people will increase and accordingly the death rate will also increase in India. Figure 20 is showing the prediction of next 2 months when lockdown will open completely and people started travelling everywhere. The prediction shows that the death numbers will increase and reach to 12,000. It means so many people will lose their lives because of coronavirus in India. As discussed above, so many people in different countries already lose their lives. This epidemic will effect Indian population very badly.

**Fig. 21:**
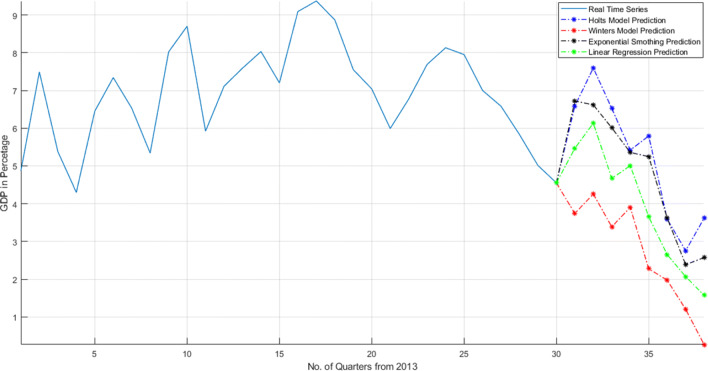
Effect on GDP of India due to COVID-19 attack and prediction of GDP for next 8 quarters

As shown in Fig. 21, the GDP of India fluctuate in every quarter of year but it will remain above than 6 from last six quarters. But due to this COVID-19, the growth of the country stops. Government took decision of lockdown and the whole country house arrest in their homes. The growth of this quarter records up to 4 and it is estimated that in 2020, the GDP will go down. The four mathematical models predict that the GDP will go down for next 8 quarters. It represents that India could not maintain its GPD for next 2 years.

**Fig. 22:**
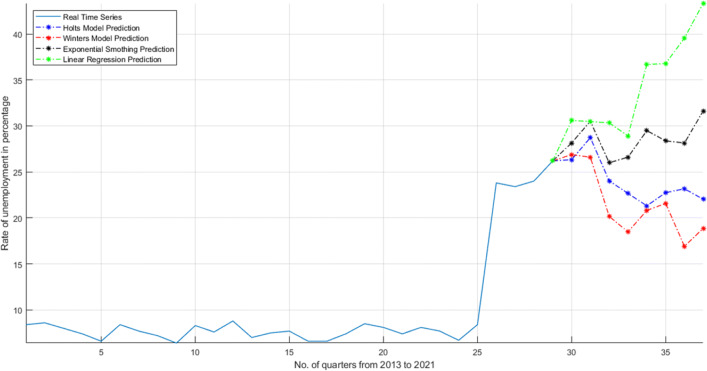
Effect on unemployment in India from 2013 to 2021. The graph shows quarter result

If the growth of the country is not going up then, unemployment problem will increase. Unemployment rate increases in 2019 and then in 2020, it increases up to 30%. Figure 22. shows that the unemployment in India will increase for next 8 quarters or for next 2 years. COVID-19 epidemic will increase the unemployment rate because every NRI citizens are coming to India again, and labours are going to their native places to leave companies. All the companies are close and no work is there. These all factors will increase the employability in India. The four mathematical model predicts that the unemployment rate will increase. Linear regression prediction shows the highest result which means, according to this mathematical modelling, the unemployment rate will increase up to 50% in India.

## COVID-19 pandemic: challenges

It has been now widely acknowledged from the impacts discussed above that the measures of the economic loss instigated by the COVID-19 pandemic will be far bigger than that affected by the 2008 global financial crisis. Therefore, based on the impact analysis, following are the broader challenges that country like India are facing and would face after the lockdown:

1. As already discussed that there is reasonable uncertainties and predictions about what and how deeper is the duration and depth of the crisis would be but one is obvious one that while, foresee the after-effects of COVID-19, there will be a huge economic policies change challenges over the coming few years. Although selected policy measures have already been proclaimed by the Government of India and the Reserve Bank of India (RBI), they are nonetheless the short-term (interim) methods and are not optimal one to recover the country economics. Therefore, it is one of the major challenges that India is going to face in coming months after COVID-19.
2. Most of the countries have declared to close schools, colleges, and universities to lockdown. The disaster develops the impasse policy-makers are fronting between shut down schools (decreasing contact and reduce lives) and keeping them open. The shutting of schools, colleges, and universities disturbs the teaching for students over the world. It accords with a vital assessment time, and many of the examinations have been suspended or canceled. Internal evaluations are possibly thought to be less essential, and several have been just canceled. The cost of this information deferrals the appreciation of both added potential and difficulties in learning and can have destructive long-term concerns for the children/students. Only some private schools/colleges/universities could implement online teaching approaches. Their low-income private and government school counterparts have closed for not having provision to e-learning solutions.
3. The healthcare sector is at the centric of this harass global pandemic situation, and the private sector has grown to the instance, by proposing to the government all the assistance it requires, be it testing facilities, preparation of quarantine beds, protection equipment, medical devices, medicines, ventilators, doctors nurse, and other medical staffs etc. deploying in identified nodal hospitals. Because, before this pandemic, the healthcare infrastructure was not so huge, maintained, and planned to tackle such large situations. Similarly, few testing facilities, shortage of isolation beds, protecting equipment, medicines, and the worst and lack public health care facilities are only available in country wide and therefore appear as big challenge that India yet facing to fight COVID-19.
4. As the COVID-19 pandemic remains to pound global and Indian supply chains, obstructing international business also in the development, the right endurance of Indian trade export is at stake. With foreign buyers and buying companies either withdrawing or suspending pre-confirmed export deals. Same is the situation small all industries because of lockdown and shortage of manpower, labour, and raw material, regardless of the size of trade industry (big or small scale). The downfall of the economics is the major reason of shutdown of many Indian industries all over and certainly government have to with economic experts to recover country with this closure of business.
5. As already deliberated and discuss that Indian transportation industry has never been experience such kind of fall of the time due to COVID-19 and lockdown, except essential goods transport, all type of transport services are on shut down and dealing with high revenue loss and near to deadline. When the situation come on track, it also may the one of key challenges to establish the transport sector on track subject to the constraints of further spread of COVID-19 because in country like India, wheel of economic completely relies upon its transport facility whether it is private or public.
6. In sequence with the transport sector shutdown and only normal run causes the less demand on day ahead, oil market bids and the negative price of the crude oil during this time better explain the challenges of oil industry and their respective hour by hour financial loss in India and globally.
7. Power and energy-related services also affect major participants of COVID-19 on generation, transmission, and distribution side majorly. The lockdown has instigated electricity demand to drop as industrial electricity consumption grasps a maximum portion in most countries’ consumption mix and result of high revenue loss due to day ahead commitment is unmanaged for which electricity trading companies already pay. This may also be the serious concern for Indian power ministry after lockdown period gets over.

## Recommendations and future roadmaps

Looking forward of early lockdown over, the world would need optimal planning strategies in all the driving sectors discussed above to appropriately manage huge loss of country. Based on the available literature and presented in this paper, following recommendations and suggestion have been put forward to overcome the challenges of COVID-19:

1. As far concerning the economics that is already in the worst phase of the time even before the COVID 19, now, the major concern has to pay to these uncertainties, policy reactions are expected to be responsive. Few notable threats in fiscal, monetary, and financial policies must have to be incorporated and implement precisely in the most effective way to prevent a long-term economic disaster.
2. The worldwide lockdown of educational institutes (schools. Colleges, universities) is going to basis significant disturbance in students/children’ education; interruptions in internal evaluation. To remove these to mitigation and negative impacts, educational institute necessitates resources to rebuild the damage in learning, once open. How can these resources be utilized, and how to aim the children/students who were particularly needed, is an open question? New graduates/professional/research scholars should care for their entry to the employment market to escape more prolonged unemployment.
3. Apart from, the Prime Minister of India call to respect healthcare professionals has truly been a morale booster. However, with falling revenues, government measures to support in terms of liquidity infusion, tax aids, and other disclaimers have turn into decisive for the endurance of health services suppliers of the country. Other than this, government should emphasis more inclusion on private healthcare facilities, NGOs, improve public healthcare, promote central/private/public research institute to develop low-cost healthcare medicines, protecting equipment, devices, testing facilities, place for isolation centres, accessories etc. to fight COVID-19.
4. As far concerning to the Indian industrial sector, India is need to consider to permit certain types essential of manufacturing and small or large industries to restart with some limitations to kick-start the economy and escape jobs/employment losses while the nation has world’s largest lockdown to hold the COVID-19 outbreak. Big industries, with appropriate sanitation and social distancing rules at work place, in areas such as textiles, automobiles, and electronic manufacturing should be deliberated to function with 30–40% capacity.
5. For Indian transport sector, in order to ensure a planned synchronised response and operative implementation of measures by public/private transport firms and authorities, contagious virus or pandemic response strategies shall create the origin for action and extent execution. Furthermore, all actions taken by governmental agencies, public and private transport enterprises in order to confirm safety of staff and passengers along with the disputing a further blowout of COVID-19 shall be based on inclusive impact evaluations. Moreover, some social, environmental, and climate on top of economic influences of measures shall be taken into considerations.
6. As consider Indian Oil market, fall in oil costs is an excessive boon in the long run if continued. It will deliver needed stability in the exchange market and also have encouraging impact on the balance of payments, enhancing the scenarios for the oil selling companies and the automobile sector. However, the entire world is on the side of precautions and the opportunities in hand are partial; this can be adapted into a prospect for India with the aid of a few paces at the government-policy making level.
7. In order to overcome the less demand of power supply and revenue loss of distribution companies, Indian power ministry has to be more reliant upon its renewable generation during lockdown instead the conventional large base plant supply and try to manage the market-clearing price of wholesale electricity market as well as steps need to be consider to make supply-demand balance as soon as possible.
8. Additionally, motivational ministries and its policymakers, leadership by centre, state governments, local and financial organizations will play a crucial role to promote, manage, and examine the close functionality of legal policies/guidelines/programmes implementation.
9. Most of the planning schemes by the government being unsuccessful due to improper execution of guidelines, hence a dedicated monitoring governance mandatory.
10. In most of the developing countries like India, the political influence and lack of transparency are the major causes of such critical programmes failure, thus need to be reduce.

## Conclusions

This article drew an attempt to overlook unprecedented challenges that are currently being faced by India at all fronts due to COVID 19 and also try to suggest the few points how rapidly can recover and improve on different sectors. Major of the impacts on the crucial industries that seems more accelerated one (also more affected due to COVID 19 outbreak), in driving any nation economics are thoroughly discussed with their past and present growth in the overall development and progress. These sectors majorly comprise economic and financial, educational, healthcare, industrial, power and energy, oil market, employment, and environment. COVID 19 outbreak and lockdown impacts on this important sector are examined, analyzed, and interpreted in many aspects. Based on the impacts summary and available data and forecasted data, comprehensive mathematical models, namely Holt-Winter Model, Exponential smoothing, Linear Regression, and Seasonality Linear Regression, are used to analyze the magnitude of the impacts which graphically demonstrated, which reveals the degree and level of impact of COVID 19 outbreak. All the models are tested on COVID’s patient rate, daily cases encountered, the number of deaths encountered, GDP of India, and unemployment sectors. Based on the results obtained and comparison best model is predicated i.e., Holt’s and Winter’s model. This comparative study helps in realizing the impacts of this pandemic and proposes some strategies to lessen these impacts as earliest before it harms more the country like India. This comprehensive analysis helps realize how deep the impacts are and suggest planning certain methodologies or strategies for the Indian government and policymakers to overcome these impacts as early as possible before the worst happens to the development of the Indian nation. Based on the detailed overview and impact analysis results, certain recommendations are also made and graphically illustrated, which mainly reflects the degree and impact level of COVID 19 spread in three-time duration, i.e., before, during, and after COVID 19. Based on the impacts summary, available data, and forecasted data, four inclusive mathematical model namely Holt-Winter Model, Exponential smoothing, Linear Regression, and Seasonality Linear Regression are used to analyse the gravity of the impact which graphically demonstrated, which primarily reveals the degree and level of impact of COVID 19 outbreak. All the models are tested on COVID’s patient rate, daily cases encountered, the number of deaths encountered, GDP of India, and unemployment sectors. Based on the results obtained and comparison best model for prediction is presented. This comparative study helps realize the impacts of this pandemic and proposes some strategies to lessen these impacts as earliest before it harms more the country like India. Based on the detailed overview and impact analysis results, specific recommendations are also made.
